# HAUS Augmin-Like Complex Subunit 1 Influences Tumour Microenvironment and Prognostic Outcomes in Glioma

**DOI:** 10.1155/2022/8027686

**Published:** 2022-07-12

**Authors:** Qi Yao, Xinqi Ge, Zhichao Lu, Jinlong Shi, Jianhong Shen, Jian Chen

**Affiliations:** ^1^Department of Neurosurgery, Affiliated Hospital of Nantong University, Nantong 226001, China; ^2^Department of Clinical Biobank, Affiliated Hospital of Nantong University, Nantong, China

## Abstract

**Background:**

The expression of HAUS Augmin-like complex subunit 1 (HAUS1), a protein-coding gene, is low in normal samples among various cancers with pan-cancer analysis. The depletion of HAUS1 in cells decreases the G2/M cell compartment and induces apoptosis. However, the detailed expression pattern of HAUS1 and its correlation with immune infiltration in glioma (LGG and GBM) (LGG: low-grade glioma; GBM: glioblastoma) remain unknown. Therefore, in this study, we examined the role and prognostic value of HAUS1 in glioma.

**Methods:**

Transcriptional expression data of HAUS1 were collected from the CGGA and TCGA databases. The Kaplan–Meier analysis, univariate and multivariate Cox analyses, and receiver operating characteristic (ROC) curves were used to analyse the clinical significance of HAUS1 in glioma. The STRING database was used to analyse protein-protein interactions (PPI), and the “ClusterProfiler” package was used for functional enrichment analysis to examine the possible biological roles of HAUS1. In addition, the HAUS1 promoter methylation modification was analysed using MEXPRESS, and the association between HAUS1 expression and tumour-infiltrating immune cells was investigated using CIBERSORT.

**Results:**

Based on the data retrieved from TCGA (703 samples) and CGGA (1018 samples), an elevated expression of HAUS1 was observed in glioma samples, which was associated with poorer survival of patients, unfavourable clinical characteristics, 1p/19q codeletion status, WHO grade, and IDH mutation status. Furthermore, multivariate and univariate Cox analyses revealed that HAUS1 was an independent predictor of glioma. HAUS1 expression level was associated with several tumour-infiltrating immune cells, such as Th2 cells, macrophages, and activated dendritic cells. The outcomes of ROC curve analysis showed that HAUS1 was good to prognosticate immune infiltrating levels in glioma with a higher area under the curve (AUC) value (AUC = 0.974).

**Conclusions:**

HAUS1 was upregulated and served as a biomarker for poor prognosis in patients with glioma. High HAUS1 expression was associated with several tumour-infiltrating immune cells such as Th2 cells, macrophages, and activated dendritic cells, which had high infiltration levels. Therefore, these findings suggest that HAUS1 is a potential biomarker for predicting the prognosis of patients with glioma and plays a pivotal role in immune infiltration in glioma.

## 1. Introduction

Glioma is a life-threatening highly malignant primary brain tumour with a high mortality rate [1, 2]. However, owing to diffusive and invasive growth, the treatment of glioma often leads to a dismal prognosis [3, 4]. Moreover, the overall survival of patients with glioma is poor, with the 5-year survival rate of adult patients being <5% [5, 6]. Chemotherapy resistance of glioma may be the main cause of poor prognosis [7]. Nowadays, lacking of corresponding molecular markers and understanding of the mechanism of glioma pathogenesis bring about a huge challenge to the early diagnosis of glioma [8]. Although there are some predictive molecular markers such as 1p/19q codeletion and IDH mutations, the high complexity of the molecular mechanism of glioma urgently requires more prognostic markers for glioma [9]. Therefore, it is imperative to discover new therapeutic strategies to enhance the overall survival of patients with glioma.

The correlation between immunotherapy and the tumour microenvironment (TME) has been receiving increasing attention [10]. TME plays an important role in chemotherapy, radiotherapy, and immunotherapy [11], and the efficacy of immunotherapy can be determined by TME, which differs among organs [12]. In addition, immunotherapy has been manifested to gradually become an integral component of cancer therapy [13]. Studies have revealed that analysing the characteristics of TME, especially immune cell infiltration, can improve the understanding of the likelihood of immunotherapeutic response or survival [14]. In recent years, owing to the emergence of novel immunotherapy strategies involving PD-1 and PD-L1 and the clinical efficacy of PD-1 and PD-L1 blockade in various solid cancers, blocking of the PD-1 and PD-L1 pathways has emerged as an effective strategy for cancer treatment [15, 16]. PD-L1 has been recently identified as a frequently used biomarker for anti-PD-1-based immunotherapy [17]. Moreover, clinical trials have revealed that integrating PD-1/PD-L1 with CTLA4 blockade treatment can provide improved therapeutic benefits compared to single blockade treatment [18]. However, only a small proportion of patients can benefit from anti-CTLA4 or anti-PD-1/PD-L1 immunotherapy [19]. Therefore, identifying novel immune-related biomarkers is necessary to improve the efficacy of immunotherapy.

HAUS augmin-like complex subunit 1 (HAUS1) is a protein-coding gene that belongs to the HAUS protein family that contains HAUS1–8 genes. HAUS1 encodes 1 of the 8 subunits of the 390 kDa human augmin complex, which participates in the generation of microtubules inside the mitotic spindle [20, 21]. In addition, HAUS1 may play a major role in centrosome integrity and completion of cytokinesis [22]. Microtubules are widely involved in intracellular life activities, including cell morphology maintenance, cell transport, cell migration, cell division, and cell signal transduction. Therefore, as one of the augmin complexes involved in microtubule generation, HAUS1 can participate in multiple human cell life activities. However, the role of HAUS1 in tumours remains unclear, especially, its relationship with immune infiltration and TME.

In this study, we investigated the prognostic significance of HAUS1 mRNA expression and methylation in patients with glioma using data from the Chinese Glioma Genome Atlas (CGGA) and The Cancer Genome Atlas (TCGA) databases. In addition, we evaluated the enrichment functions of HAUS1 using GSEA and investigated the correlation between HAUS1 expression and infiltration levels of different tumour-infiltrating lymphocytes (TILs).

## 2. Materials and Methods

### 2.1. Data Acquisition

**Figure 1:**
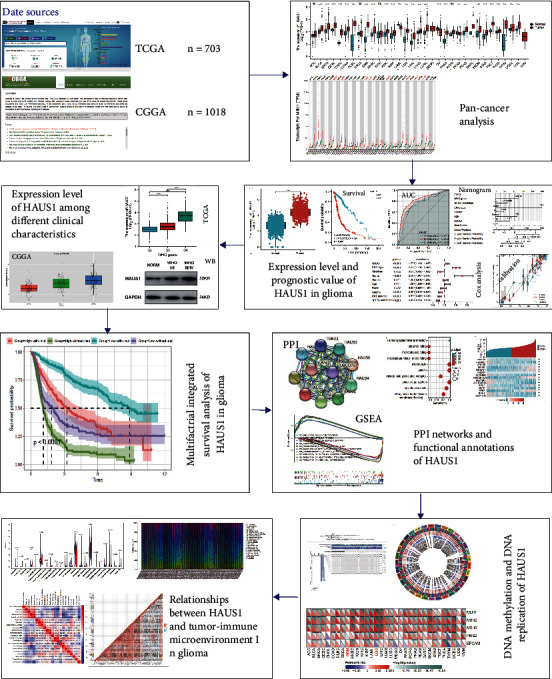
Workflow of this study.

Transcriptional RNA-sequencing data of glioma samples, including transcriptional expression data and matched clinical information, were retrieved from TCGA (https://cancergenome.nih.gov/) and CGGA (http://www.cgga.org.cn) databases [23]. A total of 703 samples were obtained from TCGA dataset, whereas 1018 samples were obtained from the CGGA dataset. The clinical parameters of the patients with complete clinical data in TCGA and CGGA databases are listed in Table 1, respectively. In addition, pan-cancer analyses of HAUS1 were conducted using TCGA dataset and GEPIA (http://gepia.cancer-pku.cn/). All downloaded samples were divided into the low- and high-expression groups based on the median HUAS1 expression. Because all data were downloaded from public open-access databases (TCGA and CGGA), it was not necessary to obtain ethics approval or informed consent.  Figure 1 shows the workflow of this study.

### 2.2. Differential Expression Analysis

GEPIA (http://gepia.cancer-pku.cn/) is an online database for gene expression profiling and interactive analyses of tumour and normal samples; it comprises 9736 tumour samples and 8587 normal samples [24, 25]. In this study, we used GEPIA to examine the expression profile of HAUS1 in glioma and conduct a pan-cancer analysis to examine HAUS1 expression level in 33 cancers.

### 2.3. Protein-Protein Interaction Analysis and Functional Enrichment Analysis

The STRING database (http://string-db.org/) is the largest database of protein-protein interactions for predicting functional relationships between proteins [26, 27]. In this study, we used the STRING database to screen for co-expression genes and build PPI networks with an interaction score of >0.4. Subsequently, we used the “ClusterProfiler” and “ggplot2” packages for Gene Ontology (GO) enrichment and Kyoto Encyclopedia of Genes and Genomes (KEGG) pathway analysis to examine the enrichment function of these genes [28].

### 2.4. Methylation Analysis of HAUS1

MEXPRESS (https://mexpress.be/) is an easy-to-use online tool for integration and visualisation of clinical expression and DNA methylation data of cancer and normal tissues from TCGA database [29, 30]. In this study, we used the MEXPRESS online tool to estimate and visualise DNA methylation of HAUS1 in TCGA dataset.

### 2.5. Analysis of Differentially Expressed Genes (DEGs)

Data retrieved from TCGA database were divided into the low- and high-expression groups based on the median HAUS1 expression level. Differentially expressed genes (DEGs) between the two groups were screened using the limma, heatmap, and ggplot2 packages in R. These DEGs were visualised on a volcano map, and the top 15 genes were visualised on heat maps. The cut-off values for screening DEGs were set as |log 2 fold change| > 1.5 and adjusted *P* value <0.001.

### 2.6. Functional Enrichment Analysis

GSEA was performed to investigate the biological pathways that differed substantially between the high- and low-HAUS1-expression groups. The “c2.cp.kegg.v7.4.symbols.gmt” and “c5.all.v7.4.symbols.gmt” gene sets were used to evaluate biological processes related to HAUS1. The analysis was performed using 1000 permutations, and *P*- value <0.05 and (FDR) *q* value <0.05 were set as the cut-off criteria.

### 2.7. CIBERSORT Analysis

The CIBERSORT algorithm was used to examine the association between the expression of HAUS1 and the infiltration of 22 types of TILs in glioma. CIBERSORT is an online tool that uses a deconvolution algorithm to sensitively and specifically calculate the proportion of TILs [31, 32]. We divided CGGA samples into the low- and high-expression groups according to the median expression level of HAUS1.

### 2.8. Quantitative RT-PCR

Total RNA was isolated from the paraneoplastic and tumour tissues of patients with glioma of different grades using the TRIzol reagent (Sigma-Aldrich, St. Louis, MO, USA). RNA from each sample (2 *μ*g) was reverse transcribed to cDNA, which was used as a template in a 20-*μ*L reaction mixture (10 *μ*L of PCR mixture, 0.5 *μ*L of forward and reverse primers each, 2 *μ*L of cDNA template, and an appropriate volume of water). Subsequently, quantitative reverse transcription polymerase chain reaction (qRT-PCR) was performed using FastStart Universal SYBR ®Green Master (Roche, Germany) on an ABI QuantStudio5 Q5 real-time PCR System (Thermo Fisher Scientific, USA). The PCR conditions were maintained as follows: initial DNA denaturation at 95°C for 30 s, followed by 45 cycles at 94°C for 15 s, 56°C for 30 seconds, and 72°C for 20 seconds. Each sample was examined in triplicate. The threshold cycle (CT) readings were recorded and normalised to glyceraldehyde 3-phosphate dehydrogenase (GAPDH) levels in all samples using the 2^−ΔΔCT^ method. The mRNA expression levels of samples were compared to those of para-cancerous tissue controls. The sequences of primer pairs for target genes are shown in Table 2.

### 2.9. Western Blot

Paraneoplastic tissue and tumour tissue samples from patients with glioma of different grades were lysed in RIPA buffer (Solarbio, Beijing, China) supplemented with protease and phosphatase inhibitors for 30 min, followed by denaturation at 95°C for 10 min. The protein samples were then separated via SDS-PAGE and transferred onto polyvinylidene fluoride (PVDF) membranes. Subsequently, the membranes were blocked with 5% skim milk powder solution for 1 h and incubated overnight with primary antibodies, including anti-HAUS1 antibody (1 : 500, Abclonal, A17250) and anti-GAPDH antibody (1 : 5000, Abcam, ab8245). The following day, the membranes were incubated with secondary antibodies (1 : 5000) for 2 h at room temperature. Thereafter, an ECL kit (Billerica Millipore, MA, USA) was used for observation, and protein bands were visualised on a ChemiDoc detection system (Bio-Rad, Hercules, CA, USA) and quantified using the ImageJ software (National Institutes of Health, USA).

### 2.10. Statistical Analysis

Statistical analyses of TCGA and CGGA data were conducted using various R packages (version 3.6.1). The relationship between HAUS1 and different clinical characteristics was evaluated using logistic regression. In addition, multivariate and univariate Cox analyses were performed to analyse the correlation between different clinical parameters and HAUS1 expression. The results of Cox analyses are presented in Table 3. The Kaplan–Meier curve revealing progression-free interval (PFI), disease-specific survival (DSS), and overall survival (OS) was constructed using the survival package in R, and time-dependent receiver operating characteristic (ROC) curves were constructed using the survival ROC package. Statistical significance was set as *P* < 0.05.

## 3. Results

### 3.1. Pan-cancer Analysis of the Expression Pattern and Prognostic Significance of HAUS1

**Figure 2:**
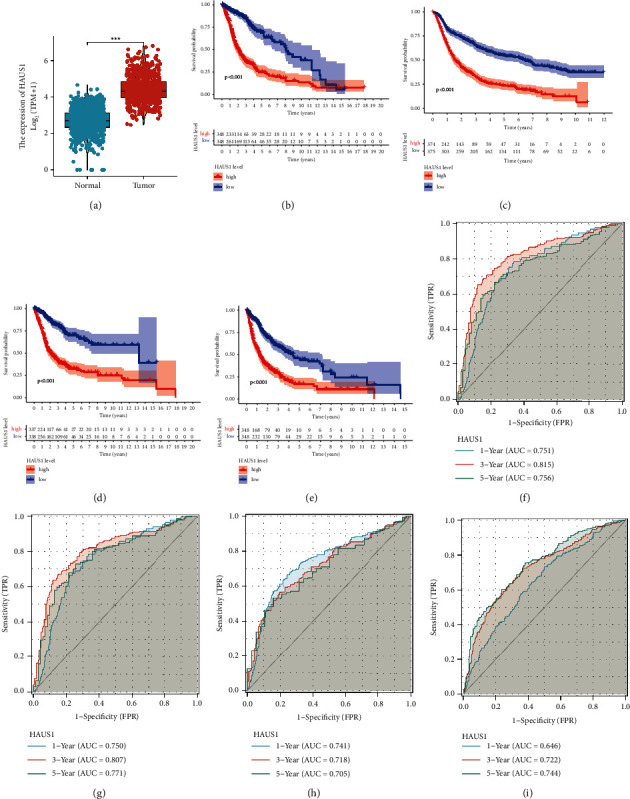
Expression level and prognostic significance of HAUS1 in glioma. (a) Expression level of HAUS1 in glioma and normal tissues based on the GTEx and TCGA databases. Kaplan–Meier survival curves of (b) OS based on GTEx and TCGA databases and (c) OS based on the CGGA database. Kaplan–Meier survival curves of (d) DSS and (e) PFI based on the GTEx and TCGA databases. ROC curves demonstrate that the AUC values for predicting 1-, 3-, and 5-year (f) OS of patients are 0.751, 0.815, and 0.756, respectively, (g) disease-specific survival of patients are 0.750, 0.807, and 0.771, respectively, and (h) progression-free interval of patients are 0.741, 0.718, and 0.705, respectively, based on TCGA and GTEx databases. (i) ROC curves reveal that the AUC values for predicting 1-, 3-, and 5-year OS of patients are 0.641, 0.716, and 0.725, respectively, based on the CGGA database.

Based on the data retrieved from TCGA and GTEx databases, we discovered that HAUS1 exhibited significantly elevated expression in a majority of human cancers, except for ACC, ESCA, KICH, MESO, PCPG, LAML, OV, PRAS, SARC, and UVM (Supplementary Figure 1A). Subsequently, we used GEPIA2 to evaluate the expression level of HAUS1 in pan cancer (Supplementary Figure 1B) and found that HAUS1 expression was significantly high in DLBC, GBM, LGG, LIHC, PAAD, SKCM, TGCT, and THYM. Therefore, these results revealed that HAUS1 expression was significantly higher in DLBC, GBM, LGG, LIHC, PAAD, SKCM, TGCT, and THYM. Owing to the overexpression of HAUS1, we further evaluated its prognostic significance in these cancers. Because GBM and LGG are types of brain tumours, their data were combined for analysis. We analysed the association between OS and HAUS1 expression in DLBC, GBM, LGG, LIHC, PAAD, SKCM, TGCT, and THYM using the GEPIA2 database. As shown in Supplementary Figures 1C–F and 2A–B and Figure 2(b), HAUS1 was significantly associated with OS in glioma (LGG and GBM) and LIHC. However, HAUS1 expression was higher and more significantly associated with OS in glioma (LGG and GBM) than in LIHC. As *K*-*M* depends on the cut-off value, and the efficiency of the test is not as high as Cox, we further investigated the prognostic value of HAUS1 in pan cancer by using the tool of Sangerbox (Supplementary Figure 2C). And the results showed that the expression level of HAUS1 plays a valuable role in glioma.

The high expression level and positive prognostic value of HAUS1 in glioma (LGG and GBM) suggested that patients with LGG and GBM might be eligible candidates for anti-HAUS1 immunotherapy. Therefore, in this study, we mainly discussed the underlying mechanisms and biological functions of HAUS1 in patients with glioma.

### 3.2. Expression Level and Prognostic Value of HAUS1 in Glioma Samples from TCGA and CGGA

We compared the mRNA level of HAUS1 between normal and glioma tissue samples obtained from GTEx and TCGA databases. In Table 1, we can see that HAUS1 expression level in glioma patients has a prognostic value in WHO grade (*P* ≤ 0.01), IDH mutation status (*P* ≤ 0.01), and 1p/19q codeletion status (*P* ≤ 0.01) based on the CGGA and TCGA database. As shown in Figure 2(a), HAUS1 expression was higher in glioma tissues than in normal tissues (*P* < 0.001). Furthermore, to assess whether HAUS1 could serve as a prognostic marker for glioma, we performed Kaplan–Meier survival analysis to compare the PFI, DSS, and OS of patients with glioma between the low- and high-HAUS1-expression groups. In TCGA dataset, patients in the high-HAUS1-expression group had a worse OS (HR = 3.58, *P* < 0.001) (Figure 2(b)). Similarly, in the CGGA dataset, reduced expression levels of HAUS1 were correlated with a better OS (*P* < 0.001) (Figure 2(c)). In addition, elevated HAUS1 expression level was correlated with poorer DSS (HR = 3.79, *P* < 0.001) and PFI (HR = 2.72, *P* < 0.001) (Figures 2(d) and 2(e)).

Furthermore, time-dependent ROC curves were constructed to investigate the prognostic value of HAUS1 in predicting 1-, 3-, and 5-year OS, DSS, and PFI based on TCGA data. As demonstrated in Figure 2(f), the AUC values for predicting the 1-, 3-, and 5-year OS of patients with glioma were 0.751, 0.815, and 0.756, respectively. As shown in Figure 2(g), the AUC values for predicting the 1-, 3-, and 5-year DSS of patients with glioma were 0.750, 0.807, and 0.771, respectively. In addition, the AUC values for predicting the 1-, 3-, and 5-year PFI of patients with glioma were 0.741, 0.718, and 0.705, respectively (Figure 2(h)). Furthermore, based on the CGGA data, the AUC values for predicting 1-, 3-, and 5-year OS were 0.646, 0.722, and 0.744, respectively (Figure 2(i)). Therefore, these results revealed that HAUS1 is a moderately sensitive index for predicting the prognosis of glioma and can serve as an effective biomarker for glioma.

### 3.3. Creation and Verification of a Nomogram

**Figure 3:**
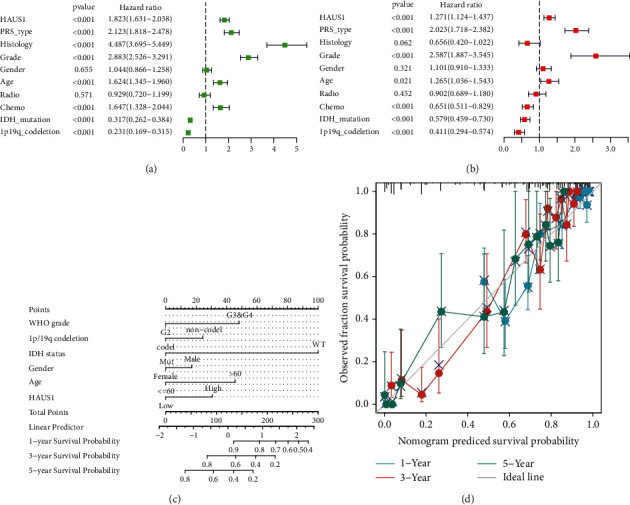
(a) Univariate Cox analysis of HAUS1 and different clinical variables. (b) Multivariate Cox analysis of HAUS1 and various clinical variables. (c) A nomogram integrating HAUS1 and other clinical variables based on TCGA database. (d) Calibration curve of the nomogram.

Multivariate and univariate Cox analyses were performed to evaluate the correlation between HAUS1 and the clinical parameters of patients with glioma. Univariate Cox analysis established certain factors that included PRS type (HR = 2.123, *P* < 0.001), histological characteristics (HR = 4.487, *P* < 0.001), grade (HR = 2.883, *P* < 0.001), age (HR = 1.624, *P* < 0.001), chemotherapy status (HR = 1.647, *P* < 0.001), isocitrate dehydrogenase (IDH) mutation status (HR = 0.317, *P* < 0.001), chromosomal arms 1p and 19q complete deletion (1p/19q codeletion status) (HR = 0.231, *P* < 0.001), and HAUS1 expression (HR = 1.823, *P* < 0.001) (Figure 3(a)). Multivariate Cox analysis established certain factors that included PRS type (HR = 1.271, *P* < 0.001), grade (HR = 2.587, *P* < 0.001), age (HR = 1.265, *P* = 0.021), chemotherapy status (HR = 0.651, *P* < 0.001), IDH mutation status (HR = 0.579, *P* < 0.001), 1p/19q codeletion status (HR = 0.411, *P* < 0.001), and HAUS1 expression (HR = 1.271, *P* < 0.001) (Figure 3(b)). A nomogram was established to assess individualised survival probability (Figure 3(c)), and calibration curves were generated to demonstrate the accuracy of the nomogram in predicting prognosis at different time points (Figure 3(d)). Altogether, these results revealed that HAUS1 could serve as an independent prognostic risk factor for glioma.

### 3.4. Relationship between HAUS1 Expression and Different Clinical Characteristics

**Figure 4:**
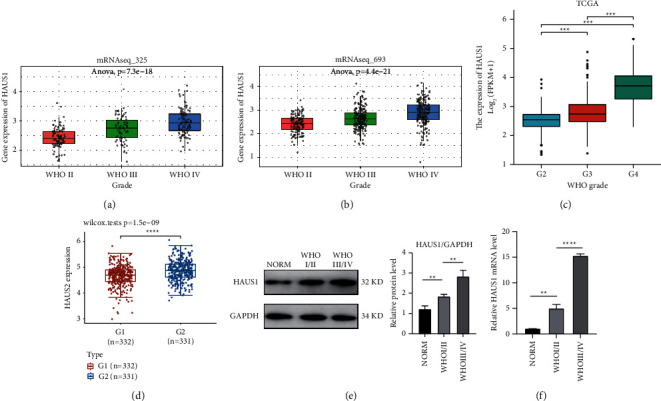
Relationship between HAUS1 expression and WHO grades (grade II, III, and IV) based on (a) mRNAseq_325 in CGGA, (b) mRNAseq_693 in CGGA, and (c) TCGA. Differences in HAUS1 expression levels among tumour samples from patients with glioma of different grades. (d) The different expression of HAUS1 in low MGMT methylation group (G1) and high MGMT methylation group (G2). (e) Representative western blot images of HAUS1 and GAPDH from three groups. GAPDH was used as a protein-loading control. Relative protein levels of HAUS1 in the three groups are expressed as mean ± S.E.M of three independent experiments (^*∗*^*P* < 0.05, ^*∗∗*^*P* < 0.01). (f) qRT-PCR results showing significant differences in mRNA expression of HAUS1; data are expressed as fold change compared to the NORM group and as mean ± SEM of three independent experiments (^*∗∗*^*P* < 0.01, ^*∗∗∗∗*^*P* < 0.0001).

A total of 325 and 693 glioma samples from the CGGA database and 749 glioma samples from TCGA database were used to assess the relationship between HAUS1 overexpression and malignant behaviour of glioma. First, we investigated the differential expression of HAUS1 in different subgroups stratified based on 1p/19q codeletion status, IDH mutation status, and WHO grade. The expression level of HAUS1 in Dataset 1 (ID: mRNAseq_325) revealed an increasing trend from LGG (Grade II) to HGG (Grade IV) (Figure 4(a)). In Dataset 2 (ID: mRNAseq_693), the expression level of HAUS1 considerably increased from Grade II to Grade IV (Figure 4(b)). Similarly, in TCGA dataset, the expression level of HAUS1 showed an increasing trend from Grade II to Grade IV (Figure 4(c)). These results suggested that advanced WHO grades were correlated with elevated expression levels of HAUS1. By analysing the relationship between HAUS1 and methylation, we found that HAUS1 was expression higher in higher MGMT methylation group (Figure 4(d)).

Furthermore, we investigated the relationship between mRNA expression of HAUS1 and WHO grades using western blot and qRT-PCR (Figures 4(e) and 4(f)). The results revealed that HAUS1 had a higher mRNA expression level in glioma tissues than in normal tissues. Moreover, the mRNA expression of HAUS1 increased with the increasing WHO grades. A high tumour grade often predicted a worse prognosis, and the mRNA expression of HAUS1 increased with the malignant progression of glioma.

**Figure 5:**
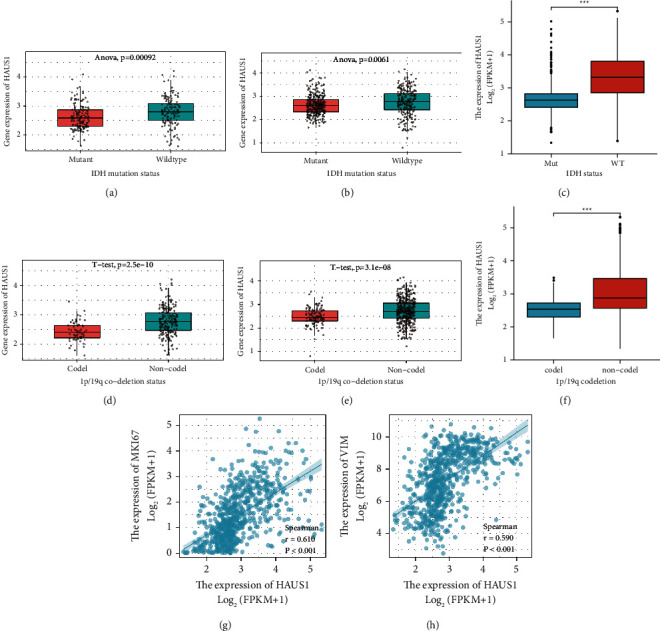
Relationship between HAUS1 expression and IDH mutation status based on (a) mRNAseq_325 in CGGA, (b) mRNAseq_693 in CGGA, and (c) TCGA and 1p/19q codeletion status based on (d) mRNAseq_325 in CGGA, (e) mRNAseq_693 in CGGA, and (f) TCGA. Correlation between HAUS1 expression level and (g) proliferation marker (Ki-67 expression) and (h) invasion marker (vimentin expression).

In addition, the mRNA expression of HAUS1 was higher in the IDH-wildtype group than in the IDH-mutant group (Figures 5(a)–5(c)); it was also high in the 1p/19q non-codeletion group (Figures 5(d)–5(f)).

Furthermore, we investigated the correlation of HAUS1 with MKI67 (Ki-67 proliferation index) and vimentin (VIM) expression level (VIM invasion index) and found that HAUS1 was strongly correlated with Ki-67 (*r* = 0.610, *P* < 0.001) (Figure 5(g)) and VIM expression (*r* = 0.590, *P* < 0.001) (Figure 5(h)). These results demonstrated that HAUS1 overexpression was related to malignant clinicopathological characteristics of glioma.

### 3.5. Multivariable Integrated Survival Analysis Based on CGGA Database

**Figure 6:**
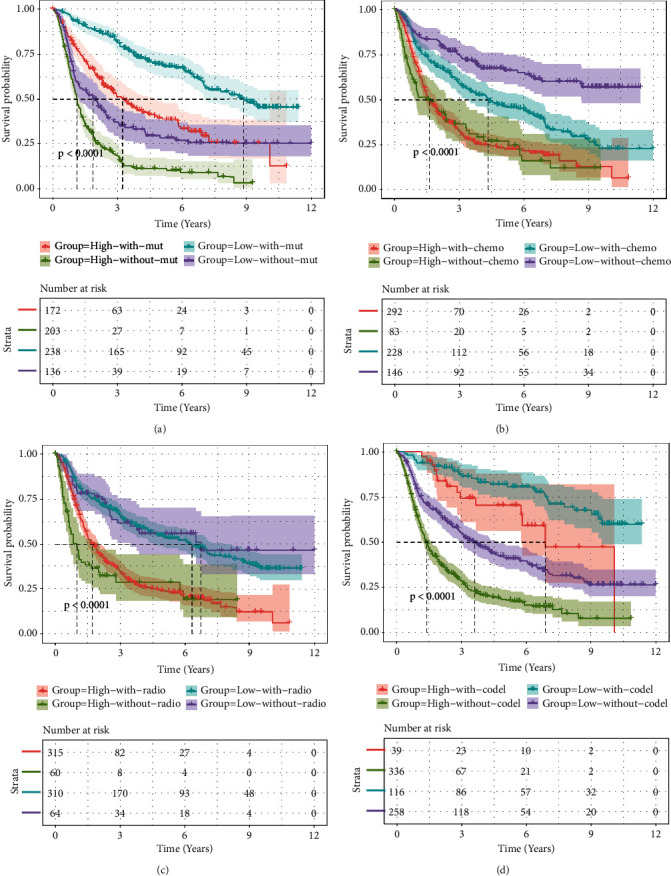
Survival analysis of patients with glioma in the high and low HAUS1 expression groups based on (a) IDH mutation status, (b) chemotherapy, (c) radiotherapy, and (d) 1p/19q codeletion status in the CGGA dataset.

To further examine the clinical value of HAUS1, we used IDH1 mutation status (Figure 6(a)), chemotherapy status (Figure 6(b)), radiotherapy status (Figure 6(c)), and 1p/19q codeletion status (Figure 6(d)) as variables for multifactorial integrated survival analysis. Elevated HAUS1 expression (red and green, Figure 6(a)) in the IDH1 mutation group indicated a dismal survival outcome, implying that HAUS1 was a significant prognostic factor for patients with glioma with the corresponding IDH1 genotypes (*P* < 0.001). Furthermore, we examined the relationship between HAUS1 expression and chemotherapy or radiotherapy. The findings revealed that lower expression levels of HAUS1 without chemotherapy indicated optimal survival outcomes (purple, Figure 6(b)), whereas higher expression levels of HAUS1 were correlated with the worst survival outcomes (red, Figure 6(b)). Therefore, these results suggested that improved survival outcomes were correlated with low-HAUS1 expression without chemotherapy. As shown in Figure 6(c), we could obviously see that low-expression level of HAUS1 with or without radiotherapy revealed better survival outcomes compared to the high-expression level of HAUS1. Finally, we examined the relationship between HAUS1 expression and 1p/19q codeletion status and found that the poorest survival outcomes were correlated with elevated HAUS1 expression without 1p/19q codeletion (green, Figure 6(d)).

### 3.6. PPI Networks and Functional Annotations

**Figure 7:**
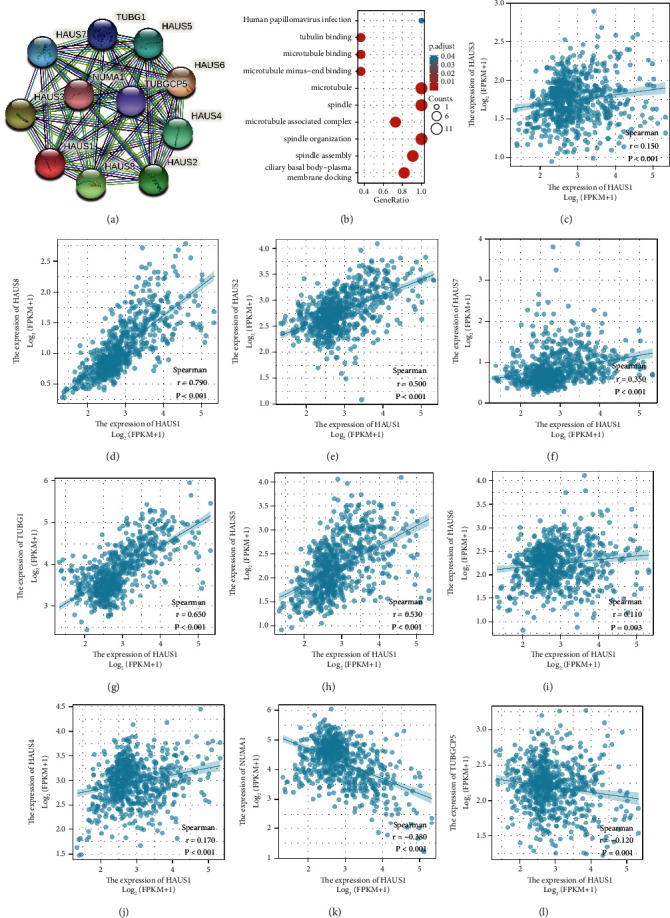
PPI networks and functional enrichment analyses. (a) A PPI network of HAUS1 and its co-expressed genes constructed using the STRING database. (b) Functional enrichment analyses of HAUS1 and its co-expressed genes. (c–l) Correlation between HAUS1 expression and its co-expressed genes.

The STRING database was used to establish PPI networks, and GO and KEGG were used for functional enrichment analyses. As shown in the PPI network in Figure 7(a), 10 co-expressed genes were closely correlated with HAUS1. As demonstrated in Figure 7(b), the biological mechanisms related to HAUS1 were associated with microtubules, spindle, and spindle organisation. The functional annotations revealed that these genes were associated with tubulin binding, microtubule binding, and microtubule minus-end binding. The relationship between HAUS1 expression and co-expressed genes in glioma based on TCGA data is demonstrated in Figures 7(c)–7(l).

### 3.7. Functional Enrichment Analysis of HAUS1

**Figure 8:**
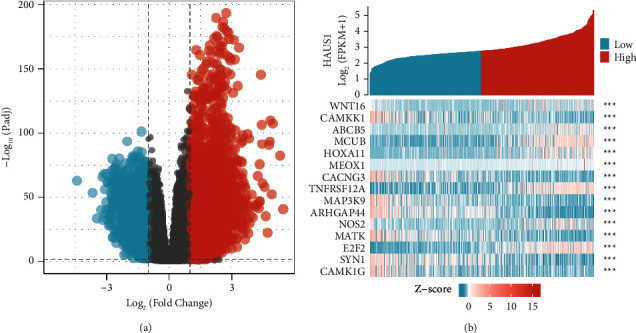
(a) Volcano plot of differentially expressed genes (DEGs) in TCGA dataset. (b) Heat map of DEGs in TCGA dataset.

**Figure 9:**
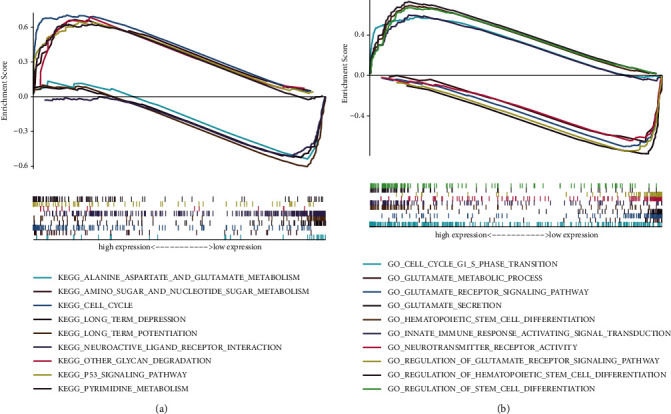
Functional enrichment analyses of HAUS1 using (a) KEGG and (b) GO analysis in TCGA dataset.

After analysing the correlation between HAUS1 and its co-expressed genes, we performed functional enrichment analysis in the low- and high-HAUS1-expression groups. To assess the underlying mechanisms of HAUS1 in promoting tumour progression, we screened for DEGs in the low- and high-HAUS1-expression groups. A total of 1457 DEGs were identified; of which, 1203 were upregulated and 254 were downregulated. A heat map and volcano plot were used to demonstrate the expression of these DEGs (Figures 8(a) and 8(b)). Furthermore, GSEA was performed to investigate key pathways associated with HAUS1. As shown in Table 4, *P* < 0.05 and FDR < 0.05 were used as the cut-off criteria. KEGG analysis revealed five categories that were positively associated with elevated expression of HAUS1: cell cycle, P53 signalling pathway, nucleotide sugar, and amino sugar metabolism, pyrimidine metabolism, and degradation of other glycans. In addition, KEGG analysis revealed four negatively correlated categories: long-term depression; neuroactive ligand–receptor interaction; long-term potentiation and alanine, aspartate, and glutamate metabolism (Figure 9(a)). GO analysis revealed five categories that were positively correlated with high expression of HAUS1: modulation of haematopoietic stem cell differentiation, modulation of stem cell differentiation, G1–S phase transition in the cell cycle, and transduction of innate immune response-activating signal. In addition, GO analysis revealed five negatively correlated categories: neurotransmitter receptor activity, glutamate secretion, glutamate metabolism, glutamate receptor signalling pathway, and modulation of glutamate receptor signalling pathway (Figure 9(b)). These results showed that pathways regulating cell and body metabolism and amino acid catabolism, which are important for patients with glioma, were strongly associated with HAUS1 expression.

### 3.8. Relationship between HAUS1 Expression and DNA Methylation and Replication

To further examine the underlying mechanisms of HAUS1 upregulation in glioma samples, we analysed the association between HAUS1 expression and its methylation. We found a methylation site (cg06121461) in the DNA sequence of HAUS1 that was negatively correlated with its expression in GBM samples (Supplementary Figure 3A). In LGG samples, we found six methylation sites (cg18069568, cg20479805, cg26626598, cg21686188, cg26713775, and cg04376617) that were negatively correlated with HAUS1 expression (Supplementary Figure 3B). Furthermore, we performed differential expression analysis of four DNA methyltransferases (DNMT1, DNMT2, DNMT3A, and DNMT3B) and assessed their correlation with HAUS1 expression by using Sangerbox tool. As demonstrated in Supplementary Figure 3C, HAUS1 expression was positively and strongly associated with the four DNA methyltransferases (DNMT1: *R* = 0.73, *P* = 1.7*e* − 87; DNMT2: *R* = 0.43, *P* = 8.3*e* − 25; DNMT3A: *R* = 0.56, *P* = 3.3*e* − 45; DNMT3B: *R* = 0.67, *P* = 5*e* − 68) in LGG samples, whereas, it was positively correlated with only DNMT2 (*R* = 0.22, *P* = 0.014) in GBM samples. In addition, we evaluated the association between HAUS1 expression and DNA replication. Mismatch repair (MMR) is a well-recognised intracellular mechanism. When a critical gene function in MMR is lost, it leads to errors in DNA replication that cannot be repaired, resulting in the production of a large number of somatic mutations. In this study, we used TCGA expression profile data to assess the association between HAUS1 expression and mutations of five MMR genes, namely, EPCAM, PMS2, MLH1, MSH2, and MSH6. In GBM samples, HAUS1 expression was significantly positively correlated with MLH1 and MSH2. In LGG samples, HAUS1 expression was positively and strongly correlated with PMS2, MLH1, MSH2, and MSH6 and negatively correlated with EPCAM (Supplementary Figure 3D). These results indicated that HAUS1 could mediate tumorigenesis and progression of glioma.

### 3.9. Relationship between HAUS1 Expression and Tumour Immune Microenvironment in Glioma

**Figure 10:**
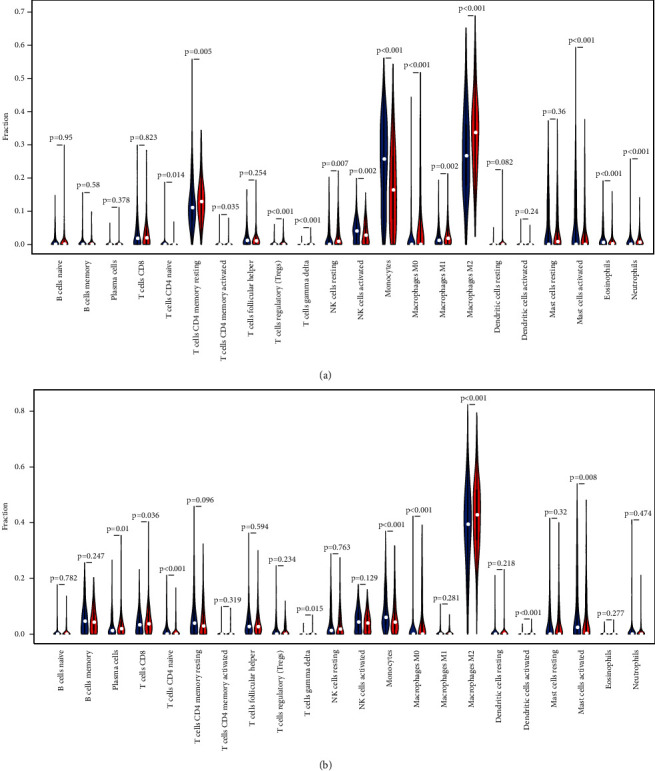
The proportion of 22 types of tumour-infiltrating lymphocytes in the high- and low-HAUS1-expression groups in (a) TCGA and (b) CGGA datasets. Red represents elevated expression, and blue represents reduced expression.

**Figure 11:**
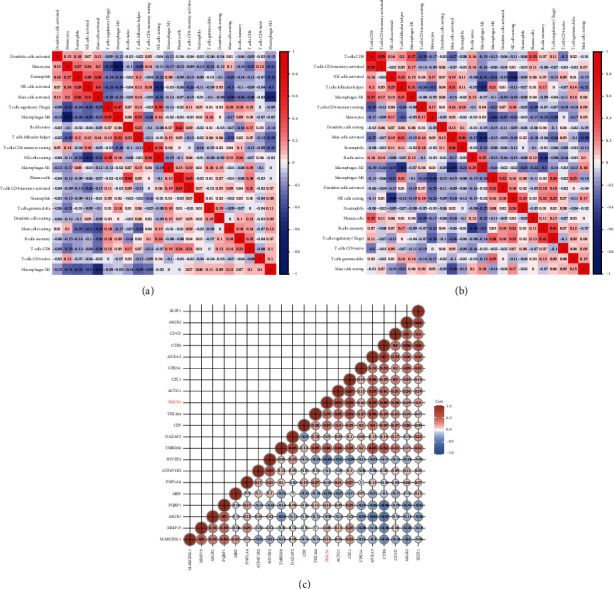
Heatmap of 22 types of tumour-infiltrating lymphocytes based on (a) TCGA and (b) CGGA. (c) The association between HAUS1 expression level and various monocytes markers.

TIL, a vital component of TME, plays a pivotal role in modulating tumour growth and progression. We used CIBERSORT to extract and process the signature gene expression profiles of immune cells to create a systematic representation of immune cell patterns. A total of 22 types of TILs were screened using TCGA and CGGA samples, and their relationship with HAUS1 expression in patients with glioma was further assessed (Supplementary Figure 4A–B). In addition, we investigated whether the tumour-immune microenvironment was different between patients with elevated HAUS1 expression and those with low-HAUS1 expression. The 689 glioma samples from TCGA and 749 glioma samples from CGGA were divided into the high- and low-expression groups based on the median HAUS1 expression. The infiltration levels of M0 macrophages, M2 macrophages, and gammadelta T cells were higher in the high-expression group than in the low-expression group, whereas, those of monocytes were lower in TCGA and CGGA datasets (Figures 10(a) and 10(b)). In addition, we investigated the relationship among the 22 types of TILs (Figures 11(a) and 11(b)). The heat map revealed a weak-to-moderate correlation among the ratios of various types of immune cells. HAUS1 was negatively correlated with monocytes; therefore, we subsequently examined the relationship between HAUS1 expression and marker genes of monocytes (Figure 11(c)). The findings revealed that HAUS1 was negatively associated with HIVEP2 (*R* = −0.43) and MBP (*R* = −0.29).

**Figure 12:**
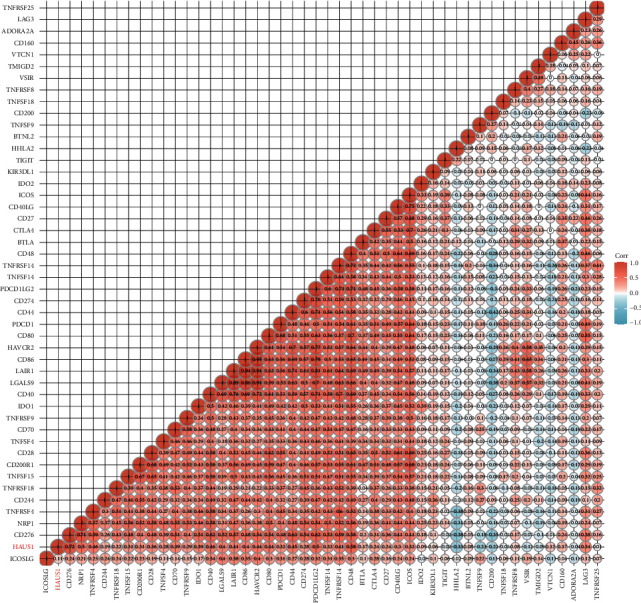
Relationship between HAUS1 expression and immune checkpoints.

In addition, as previous studies have confirmed that the infiltration of immune cells in TME is closely correlated with checkpoint blockade therapy [33], we investigated the association between the expression of HAUS1 and distinct immune checkpoint molecules (Figure 12). The results showed that most immune checkpoints examined were positively correlated with HAUS1 expression, such as CD276 (*R* = 0.72), CD48 (*R* = 0.58), TNFRSF4 (*R* = 0.46), CD40 (*R* = 0.46), CD80 (*R* = 0.44), and HAVCR2 (*R* = 0.44), whereas HHLA2 (*R* = −0.30), TNFSF9 (*R* = −0.23), and CD200 (*R* = −0.23) were negatively correlated with HAUS1 expression.

Finally, to investigate the predictive performance of HAUS1 based on the infiltration levels of immune cells in glioma, ROC curves were generated to compare the AUC values of HAUS1, PD-L1, CTLA-4, and Siglec15. The results showed that the predictive power of HAUS1 was higher (AUC = 0.974, 95% CI = 0.968–0.981) than that of the other markers (Supplementary Figure 5A–D).

## 4. Discussion

Gliomas are highly heterogeneous tumours, including low-grade glioma (LGG) to high-grade glioma (HGG), and are the most common primary central nervous system (CVS) tumours in humans [34, 35]. Based on the classification proposed by the World Health Organization (WHO), glioma can be classified as astrocytoma, oligodendroglioma, or mixed oligoastrocytoma [34]. Although multiple clinical treatments are available for glioma, such as radiotherapy, surgery, oral medication, and chemotherapy, the prognosis of patients remains unsatisfactory [36]. Moreover, currently available therapeutic interventions are known to exhibit severe short-term and long-term side effects, such as neurocognitive deficits, sterility, endocrinopathies, and postoperative mutism. Furthermore, patients with recurring illnesses have a notably unsatisfactory prognosis after first therapy, with a median survival of <6 months [37]. Therefore, developing more efficient treatment methods for glioma is necessary. Previous studies have shown that glioma cells may act against various constituents in their microenvironment, resulting in the establishment of an immunosuppressive microenvironment and the progression of glioma [38]. In this context, modulating the glioma microenvironment while targeting glioma cells with key biomarkers simultaneously appears to be a feasible therapeutic approach to treat patients with glioma [39].

In this study, we discovered that the mRNA expression of HAUS1 was higher in glioma tissues than in normal tissues and increased with tumour grade. However, little information is available regarding the association between HAUS1 expression and glioma risk and prognosis. The pan-cancer analysis in this study revealed that HAUS1 expression was high in most cancers but only had a high statistical significance in the glioma tissue samples. We found that HAUS1 overexpression was associated with PRS type, histological characteristics, age, grade, 1p/19q codeletion status, chemotherapy status, and IDH mutation status. Therefore, we speculated that HAUS1 overexpression might be correlated with the malignant behaviour of glioma. Furthermore, multivariate and univariate Cox analyses confirmed that HAUS1 is an independent prognostic factor for glioma. The Kaplan–Meier curves demonstrated that the elevated HAUS1 expression was associated with a shorter OS, PFI, and DSS, and ROC curves demonstrated that HAUS1 might serve as an effective diagnostic biomarker for the differentiation of glioma from healthy tissues. Given that HAUS1 has a good prognostic value, we built a nomogram that integrated HAUS1 expression with various clinical characteristics, and the results revealed that HAUS1 could accurately predict the 1-, 3-, and 5-year survival of patients with glioma. The presence or absence of the 1p/19q codeletion and IDH mutation were proposed as a novel glioma classification approach by the WHO [40]. Therefore, in this study, we investigated the correlation of HAUS1 with 1p/19q codeletion status and IDH mutation status. Moreover, the correlation of HAUS1 expression with chemotherapy and radiotherapy was also investigated. Functional enrichment analysis revealed that elevated HAUS1 expression was associated with cell cycle, P53 signalling pathway, and regulation of stem cell differentiation, which was consistent with the abovementioned results. These findings suggested that HAUS1 served as a prognostic biomarker for glioma.

HAUS1 is a protein-coding gene that is involved in microtubule nucleation, which is important in assembling mitotic spindle, and regulates NuMA, a crucial protein in spindle orientation [41]. In addition, HAUS1 plays a significant role in the maintenance of centrosome integrity and completion of cytokinesis [22]. In this study, we found that HAUS1 plays a crucial role in cell division and functional activity of cells. However, high HAUS1 expression was correlated with a poor OS in the patients with glioma. Therefore, we speculated that high HAUS1 expression stimulated tumour cell division and survival, thus exacerbating the malignant behaviour of glioma.

Furthermore, we investigated the correlation of HAUS1 expression with the TME and immunotherapy. HAUS1 overexpression was correlated with high infiltration of gammadelta T cells, M0 macrophages, and M2 macrophages but with low infiltration of monocytes. Monocytes are major cells of the innate immune system and play a crucial role in adaptive immune response [42, 43]. In addition, they play a significant role in TME and are correlated with cancer progression, immune escape and initiation, and regulation of immune and inflammatory responses [44, 45]. Previous studies have reported that monocytes are used as immune targets in arterial hypertension and act as pivotal immune cells in sepsis [46, 47]. In glioma, the most infiltrating immune cells are monocytes, along with microglia, accounting for approximately one-third of all the immune cells in glioma tissues [48].

Therefore, we investigated the relationship between HAUS1 and glioma. However, this study has a few limitations. First, we did not further explore and verify the functional role of HAUS1 in the immune microenvironment of glioma. However, this subject is rather novel and warrants further investigation. Second, no clinical samples were used to verify the results. However, we analysed and verified the results based on a large amount of data from public databases and in vivo, which may provide guidance and a reference for future studies.

## Figures and Tables

**Table 1:** Relationship between HAUS1 and different clinical characteristics based on the TCGA and CGGA data.

Characteristic	TCGA			CGGA		
	Low expression of HAUS1	High expression of HAUS1	*P*	Low expression of HAUS1	High expression of HAUS1	*P*
*n*	348	348		375	374	
WHO grade, *n* (%)			<0.001			<0.001
G2	174	50		173	45	
G3	126	117		119	121	
G4	10	158		83	208	
IDH status, *n* (%)			<0.001			<0.001
WT	52	194		136	203	
Mut	293	147		239	171	
1p/19q codeletion, *n* (%)			<0.001			<0.001
Codel	134	37		117	38	
Non-codel	214	304		258	336	
Gender, *n* (%)			0.193			<0.001
Female	158	140		149	158	
Male	190	208		226	216	

**Table 2:** The sequences of primer pairs for target genes.

Gene	Forward primer sequence (5′-3′)	Reverse primer sequence (5′-3′)
HAUS1	AGGAGCAACTTTCAGCCAGAG	AGCAAGAGACGGATTCGGCAT
GAPDH	AATGGGCAGCCGTTAGGAAA	GCCCAATACGACCAAATCAGAG

**Table 3:** Univariate and multivariate Cox analyses between HAUS1 and different clinical features.

Characteristics	Univariate analysis				Multivariate analysis			
	HR	HR.95L	HR.95H	*P* value	HR	HR.95L	HR.95H	*P*-value
HAUS1	1.823	1.631	2.038	<0.001	1.271	1.124	1.437	<0.001
PRS type	2.123	1.818	2.478	<0.001	2.023	1.718	2.382	<0.001
Histological characteristics	4.487	3.695	5.449	<0.001	0.656	0.420	1.022	0.062
Grade	2.883	2.526	3.291	<0.001	2.587	1.887	3.545	<0.001
Sex	1.044	0.866	1.258	0.655	1.101	0.910	1.333	0.321
Age	1.624	1.345	1.960	<0.001	1.265	1.036	1.543	0.021
Radiotherapy	0.929	0.720	1.199	0.571	0.902	0.689	1.180	0.452
Chemotherapy	1.647	1.328	2.044	<0.001	0.651	0.511	0.829	0.001
IDH mutation	0.317	0.262	0.384	<0.001	0.579	0.459	0.730	<0.001
1p/19q codeletion	0.231	0.169	0.315	<0.001	0.411	0.294	0.574	<0.001

**Table 4:** Different pathways significantly associated with high- and low-expression levels of HAUS1.

Gene set name	NES	NOM *P* value	FDR *q* value
*High expression*			
KEGG_CELL_CYCLE	2.129	<0.001	0.015
KEGG_P53_SIGNALING_PATHWAY	2.127	<0.001	0.008
KEGG_PYRIMIDINE_METABOLISM	2.067	<0.001	0.008
KEGG_AMINO_SUGAR_AND_NUCLEOTIDE_SUGAR_METABOLISM	2.047	<0.001	0.007
KEGG_OTHER_GLYCAN_DEGRADATION	1.732	0.012	0.047
GO_REGULATION_OF_STEM_CELL_DIFFERENTIATION	2.214	<0.001	0.033
GO_REGULATION_OF_HEMATOPOIETIC_STEM_CELL_DIFFERENTIATION	2.162	<0.001	0.026
GO_HEMATOPOIETIC_STEM_CELL_DIFFERENTIATION	2.149	<0.001	0.025
GO_CELL_CYCLE_G1_S_PHASE_TRANSITION	2.143	<0.001	0.022
GO_INNATE_IMMUNE_RESPONSE_ACTIVATING_SIGNAL_TRANSDUCTION	2.113	<0.001	0.022

*Low expression*			
KEGG_LONG_TERM_POTENTIATION	−1.919	0.006	0.042
KEGG_NEUROACTIVE_LIGAND_RECEPTOR_INTERACTION	−1.910	0.004	0.035
KEGG_LONG_TERM_DEPRESSION	−1.907	0.004	0.028
KEGG_ALANINE_ASPARTATE_AND_GLUTAMATE_METABOLISM	−1.855	0.002	0.039
GO_NEUROTRANSMITTER_RECEPTOR_ACTIVITY	−2.140	<0.001	0.017
GO_GLUTAMATE_SECRETION	−2.114	<0.001	0.015
GO_GLUTAMATE_METABOLIC_PROCESS	−2.114	<0.001	0.013
GO_GLUTAMATE_RECEPTOR_SIGNALING_PATHWAY	−2.101	<0.001	0.014
GO_REGULATION_OF_GLUTAMATE_RECEPTOR_SIGNALING_PATHWAY	−2.082	<0.001	0.016

## Data Availability

The data used to support the findings of this study are included in the article.

## Abbreviations

TCGA: The Cancer Genome Atlas
CGGA: Chinese Glioma Genome Atlas
GEPIA: Gene expression profiling interactive analysis
HAUS1: HAUS augmin-like complex subunit 1
GBM: Glioblastoma
LGG: Low-grade glioma
1p/19q: Chromosome arms 1p and 19q
IDH: Isocitrate dehydrogenase
PFI: Progression-free interval
DSS: Disease-specific survival
OS: Overall survival
WHO: World Health Organization
ACC: Adrenocortical carcinoma
ESCA: Esophageal carcinoma
KICH: Kidney chromophobe
MESO: Mesothelioma
PCPG: Pheochromocytoma and paraganglioma
LAML: Acute myeloid leukemia
OV: Ovarian serous cystadenocarcinoma
PRAD: Prostate adenocarcinoma
SARC: Sarcoma
UVM: Uveal melanoma
DLBC: Lymphoid neoplasm diffuse large B-cell lymphoma
GBM: Glioblastoma multiforme
LGG: Brain lower grade glioma
LIHC: Liver hepatocellular carcinoma
PAAD: Pancreatic adenocarcinoma
SKCM: Skin cutaneous melanoma
TGCT: Testicular germ cell tumours
THYM: Thymoma.

## Supplementary Materials

Supplementary Materials Supplementary Figure 1: pan-cancer analysis of HAUS1 based on (A) GTEx and TCGA databases and (B) GEPIA2. Survival curve analysis of HAUS1 expression and (C) DLBC, (D) LIHC, (E) PAAD, and (F) SKCM. Supplementary Figure 2: survival curve analysis of HAUS1 expression and (A) TGCT, (B) THYM. (C) Cox analysis of OS in HAUS1 expression in pan cancer. Supplementary Figure 3: the methylation site of HAUS1 DNA sequence associated with gene expression was revealed using MEXPRESS in (A) GBM and (B) LGG samples. (C) Relationship between HAUS1 expression and four methyltransferases. Red represents DNMT1, blue represents DNMT2, green represents DNMT3a, and purple represents DNMT3b. (D) Correlation between HAUS1 expression and five MMR genes (EPCAM, PMS2, MLH1, MSH2, and MSH6). Supplementary Figure 4: the landscape of 22 types of tumour-infiltrating lymphocytes in glioma samples in (A) TCGA and (B) CGGA datasets. Supplementary Figure 5: predictive powers for glioma infiltration between (A) HAUS1 and common markers, such as (B) PD-L1, (C) CTLA-4, and (D) Siglec15.

## References

[1] Nangami G. N., Sakwe A. M., Izban M. G. (2016). Fetuin-A (alpha 2HS glycoprotein) modulates growth, motility, invasion, and senescence in high-grade astrocytomas. *Cancer Medicine*.

[2] Tan J., Duan X., Zhang F. (2020). Theranostic nanomedicine for synergistic chemodynamic therapy and chemotherapy of orthotopic glioma. *Advanced Science*.

[3] Berberich A., Bartels F., Tang Z. (2020). LAPTM5-CD40 crosstalk in glioblastoma invasion and temozolomide resistance. *Frontiers in Oncology*.

[4] Xu R., Shimizu F., Hovinga K. (2016). Molecular and clinical effects of notch inhibition in glioma patients: a phase 0/I trial. *Clinical Cancer Research*.

[5] Ding X., Han X., Yuan H., Zhang Y., Gao Y. (2020). The impact of PPARD and PPARG polymorphisms on glioma risk and prognosis. *Scientific Reports*.

[6] Liu C., Chen J., Zhu Y. (2018). Highly sensitive MoS (2)-indocyanine green hybrid for photoacoustic imaging of orthotopic brain glioma at deep site. *Nano-Micro Letters*.

[7] Suk K. (2012). Proteomic analysis of glioma chemoresistance. *Current Neuropharmacology*.

[8] Wu Q., Wang Y., Wang H. (2018). DNA aptamers from whole-cell SELEX as new diagnostic agents against glioblastoma multiforme cells. *Analyst*.

[9] Feng X., Zhang L., Ke S. (2020). High expression of GPNMB indicates an unfavorable prognosis in glioma: combination of data from the GEO and CGGA databases and validation in tissue microarray. *Oncology Letters*.

[10] Liu L., Yang Y., Duan H. (2021). CHI3L2 is a novel prognostic biomarker and correlated with immune infiltrates in gliomas. *Frontiers in Oncology*.

[11] Huizing F. J., Garousi J., Lok J. (2019). CAIX-targeting radiotracers for hypoxia imaging in head and neck cancer models. *Scientific Reports*.

[12] Landi L., D’Incà F., Gelibter A. (2019). Bone metastases and immunotherapy in patients with advanced non-small-cell lung cancer. *Journal for ImmunoTherapy of Cancer*.

[13] Soh J. S., Jo S. I., Lee H. (2019). Immunoprofiling of colitis-associated and sporadic colorectal cancer and its clinical significance. *Scientific Reports*.

[14] Tremblay M.-L., O’Brien-Moran Z., Rioux J. A. (2020). Quantitative MRI cell tracking of immune cell recruitment to tumors and draining lymph nodes in response to anti-PD-1 and a DPX-based immunotherapy. *OncoImmunology*.

[15] Kyi C., Postow M. A. (2016). Immune checkpoint inhibitor combinations in solid tumors: opportunities and challenges. *Immunotherapy*.

[16] Wang Y., Liu X., Zou X. (2021). Metabolism and interspecies variation of IMMH-010, a programmed cell death ligand 1 inhibitor prodrug. *Pharmaceutics*.

[17] Kikuchi M., Clump D. A., Srivastava R. M. (2017). Preclinical immunoPET/CT imaging using Zr-89-labeled anti-PD-L1 monoclonal antibody for assessing radiation-induced PD-L1 upregulation in head and neck cancer and melanoma. *OncoImmunology*.

[18] Ma W.-J., Wang X., Yan W.-T. (2018). Indoleamine-2, 3-dioxygenase 1/cyclooxygenase 2 expression prediction for adverse prognosis in colorectal cancer. *World Journal of Gastroenterology*.

[19] Ye Y., Kuang X., Xie Z. (2020). Small-molecule MMP2/MMP9 inhibitor SB-3CT modulates tumor immune surveillance by regulating PD-L1. *Genome Medicine*.

[20] Goshima G., Mayer M., Zhang N., Stuurman N., Vale R. D. (2008). Augmin: a protein complex required for centrosome-independent microtubule generation within the spindle. *Journal of Cell Biology*.

[21] Uehara R., Nozawa R. S., Tomioka A. (2009). The augmin complex plays a critical role in spindle microtubule generation for mitotic progression and cytokinesis in human cells. *Proceedings of the National Academy of Sciences of the United States of America*.

[22] Rafi S. K., Butler M. G. (2020). The 15q11.2 BP1-BP2 microdeletion (Burnside–Butler) syndrome: in silico analyses of the four coding genes reveal functional associations with neurodevelopmental disorders. *International Journal of Molecular Sciences*.

[23] Wang B., Lan T., Xiao H. (2021). The expression profiles and prognostic values of HSP70s in hepatocellular carcinoma. *Cancer Cell International*.

[24] Yang S.-H., Liu W., Peng J. (2021). High expression of RhoBTB3 predicts favorable chemothrapy outcomes in non-M3 acute myeloid leukemia. *Journal of Cancer*.

[25] Fan G., Tu Y., Wu N., Xiao H. (2020). The expression profiles and prognostic values of HSPs family members in Head and neck cancer. *Cancer Cell International*.

[26] Yu Y., Gao C., Chen Y. (2020). Copy number analysis reveal genetic risks of penile cancer. *Frontiers in Oncology*.

[27] Kong Y., Feng Z.-C., Zhang Y.-L. (2020). Identification of immune-related genes contributing to the development of glioblastoma using weighted gene co-expression network analysis. *Frontiers in Immunology*.

[28] Lu G., Zhou Y., Zhang C., Zhang Y. (2021). Upregulation of LIMK1 is correlated with poor prognosis and immune infiltrates in lung adenocarcinoma. *Frontiers in Genetics*.

[29] Kumari S., Arora M., Singh J. (2021). Molecular associations and clinical significance of RAPs in hepatocellular carcinoma. *Frontiers in Molecular Biosciences*.

[30] Chen S.-H., Lin H.-H., Li Y.-F., Tsai W.-C., Hueng D.-Y. (2021). Clinical significance and systematic expression analysis of the thyroid receptor interacting protein 13 (TRIP13) as human gliomas biomarker. *Cancers*.

[31] Wang W., Wu Q., Wang Z. (2021). Development of a prognostic model for ovarian cancer patients based on novel immune microenvironment related genes. *Frontiers in Oncology*.

[32] Liu J., Huang X., Liu H. (2021). Immune landscape and prognostic immune-related genes in KRAS-mutant colorectal cancer patients. *Journal of Translational Medicine*.

[33] Zhou J., Li X., Zhang M. (2020). The aberrant expression of rhythm genes affects the genome instability and regulates the cancer immunity in pan-cancer. *Cancer Medicine*.

[34] Song H.-R., Gonzalez-Gomez I., Suh G. S. (2010). Nuclear factor IA is expressed in astrocytomas and is associated with improved survival. *Neuro-Oncology*.

[35] Li Z., Langhans S. A (2021). In vivo and ex vivo pediatric brain tumor models: an overview. *Frontiers in Oncology*.

[36] Ding L., Wang Q., Shen M. (2017). Thermoresponsive nanocomposite gel for local drug delivery to suppress the growth of glioma by inducing autophagy. *Autophagy*.

[37] Rudin C. M., Hann C. L., Laterra J. (2009). Treatment of medulloblastoma with hedgehog pathway inhibitor GDC-0449. *New England Journal of Medicine*.

[38] Wang G., Zhou H., Tian L. (2021). A prognostic DNA damage repair genes signature and its impact on immune cell infiltration in glioma. *Frontiers in Oncology*.

[39] Wu J., Frady L. N., Bash R. E. (2018). MerTK as a therapeutic target in glioblastoma. *Neuro-Oncology*.

[40] Zhang Y., Yang X., Zhu X.-L. (2021). A novel immune-related prognostic biomarker and target associated with malignant progression of glioma. *Frontiers in Oncology*.

[41] Villegas E., Kabotyanski E. B., Shore A. N., Creighton C. J., Westbrook T. F., Rosen J. M. (2014). Plk2 regulates mitotic spindle orientation and mammary gland development. *Development*.

[42] Deschner N., Rieg S., Häberle H., Dieterich H. J. (1999). Artificial colloids influence survival rate of human monocytes. *Critical Care*.

[43] Sanmarco L. M., Eberhardt N., Bergero G. (2019). Monocyte glycolysis determines CD8+ T cell functionality in human Chagas disease. *JCI Insight*.

[44] Mancuso R. I., Olalla Saad S. T., Azambuja J. H. (2021). Artesunate switches monocytes to an inflammatory phenotype with the ability to kill leukemic cells. *International Journal of Molecular Sciences*.

[45] Goring K., Huang Y., Mowat C. (2009). Mechanisms of human complement factor B induction in sepsis and inhibition by activated protein C. *American Journal of Physiology-Cell Physiology*.

[46] Wenzel P. (2019). Monocytes as immune targets in arterial hypertension. *British Journal of Pharmacology*.

[47] Schefold J. C. (2010). Measurement of monocytic HLA-DR (mHLA-DR) expression in patients with severe sepsis and septic shock: assessment of immune organ failure. *Intensive Care Medicine*.

[48] Zemp F. J., McKenzie B. A., Lun X. (2014). Cellular factors promoting resistance to effective treatment of glioma with oncolytic myxoma virus. *Cancer Research*.

