# Supplementary figures and images for: HAUS Augmin-Like Complex Subunit 1 Influences Tumour Microenvironment and Prognostic Outcomes in Glioma
Source: J Oncol. 2022 Jul 12;2022:8027686. doi: 10.1155/2022/8027686 (PMC9296284; doi:10.1155/2022/8027686)

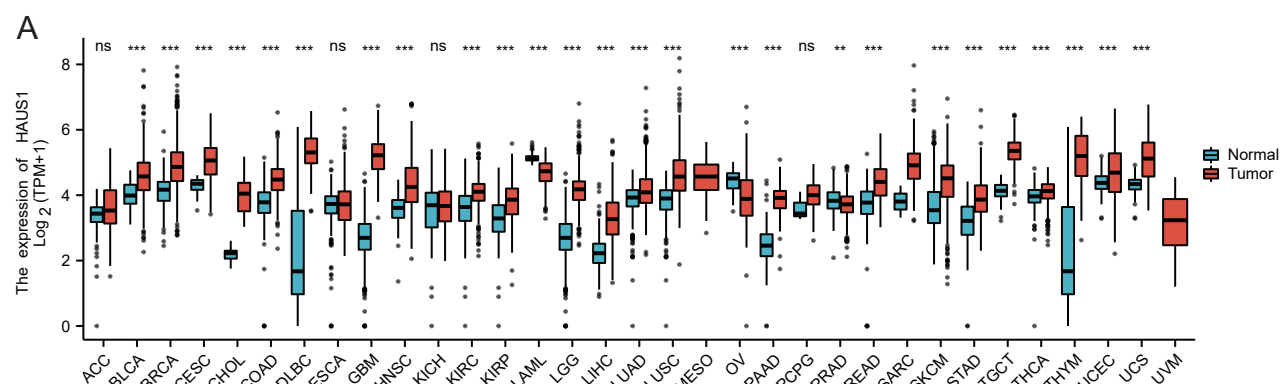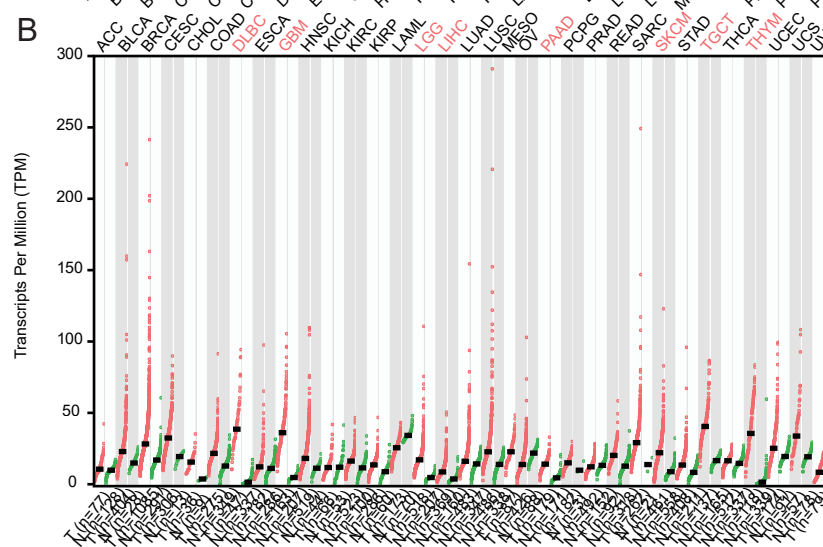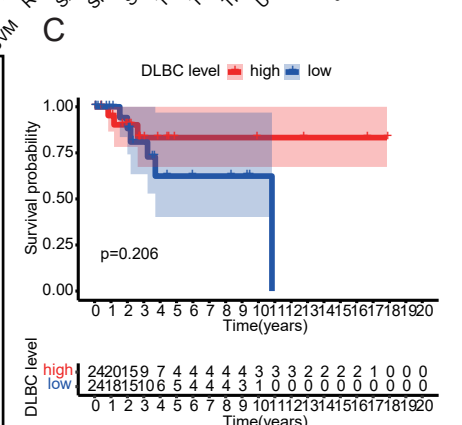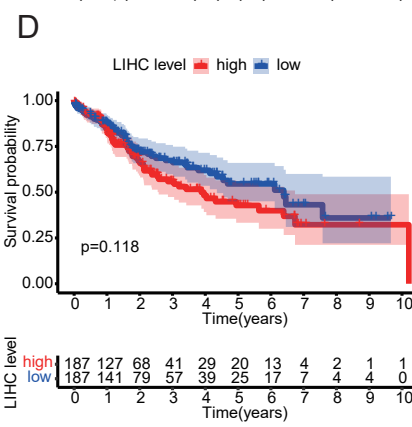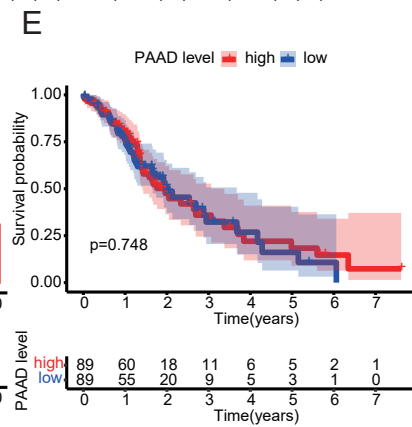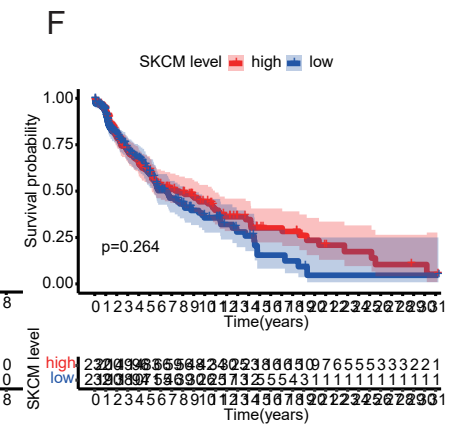

Supplement: Supplementary Materials — Supplementary Figure 1: pan-cancer analysis of HAUS1 based on (A) GTEx and TCGA databases and (B) GEPIA2. Survival curve analysis of HAUS1 expression and (C) DLBC, (D) LIHC, (E) PAAD, and (F) SKCM. Supplementary Figure 2: survival curve analysis of HAUS1 expression and (A) TGCT, (B) THYM. (C) Cox analysis of OS in HAUS1 expression in pan cancer. Supplementary Figure 3: the methylation site of HAUS1 DNA sequence associated with gene expression was revealed using MEXPRESS in (A) GBM and (B) LGG samples. (C) Relationship between HAUS1 expression and four methyltransferases. Red represents DNMT1, blue represents DNMT2, green represents DNMT3a, and purple represents DNMT3b. (D) Correlation between HAUS1 expression and five MMR genes (EPCAM, PMS2, MLH1, MSH2, and MSH6). Supplementary Figure 4: the landscape of 22 types of tumour-infiltrating lymphocytes in glioma samples in (A) TCGA and (B) CGGA datasets. Supplementary Figure 5: predictive powers for glioma infiltration between (A) HAUS1 and common markers, such as (B) PD-L1, (C) CTLA-4, and (D) Siglec15. [file 8027686.f1.zip › 8027686.f1/Supplementary Figure 1 (1).pdf]

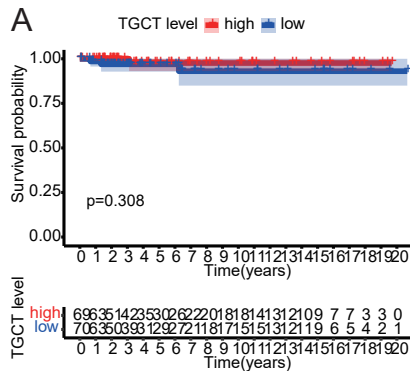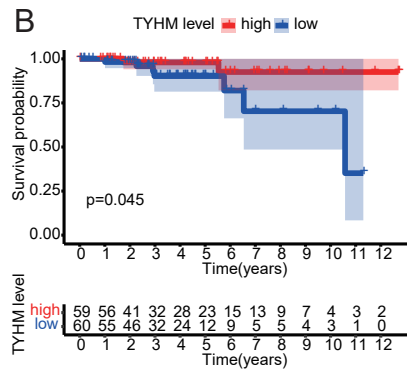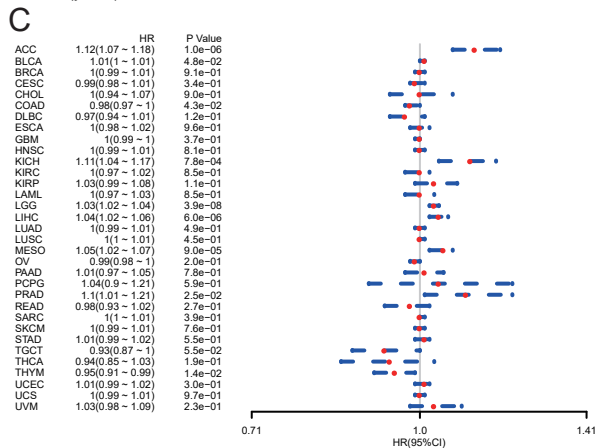

Supplement: Supplementary Materials — Supplementary Figure 1: pan-cancer analysis of HAUS1 based on (A) GTEx and TCGA databases and (B) GEPIA2. Survival curve analysis of HAUS1 expression and (C) DLBC, (D) LIHC, (E) PAAD, and (F) SKCM. Supplementary Figure 2: survival curve analysis of HAUS1 expression and (A) TGCT, (B) THYM. (C) Cox analysis of OS in HAUS1 expression in pan cancer. Supplementary Figure 3: the methylation site of HAUS1 DNA sequence associated with gene expression was revealed using MEXPRESS in (A) GBM and (B) LGG samples. (C) Relationship between HAUS1 expression and four methyltransferases. Red represents DNMT1, blue represents DNMT2, green represents DNMT3a, and purple represents DNMT3b. (D) Correlation between HAUS1 expression and five MMR genes (EPCAM, PMS2, MLH1, MSH2, and MSH6). Supplementary Figure 4: the landscape of 22 types of tumour-infiltrating lymphocytes in glioma samples in (A) TCGA and (B) CGGA datasets. Supplementary Figure 5: predictive powers for glioma infiltration between (A) HAUS1 and common markers, such as (B) PD-L1, (C) CTLA-4, and (D) Siglec15. [file 8027686.f1.zip › 8027686.f1/Supplementary Figure 2 (1).pdf]

A

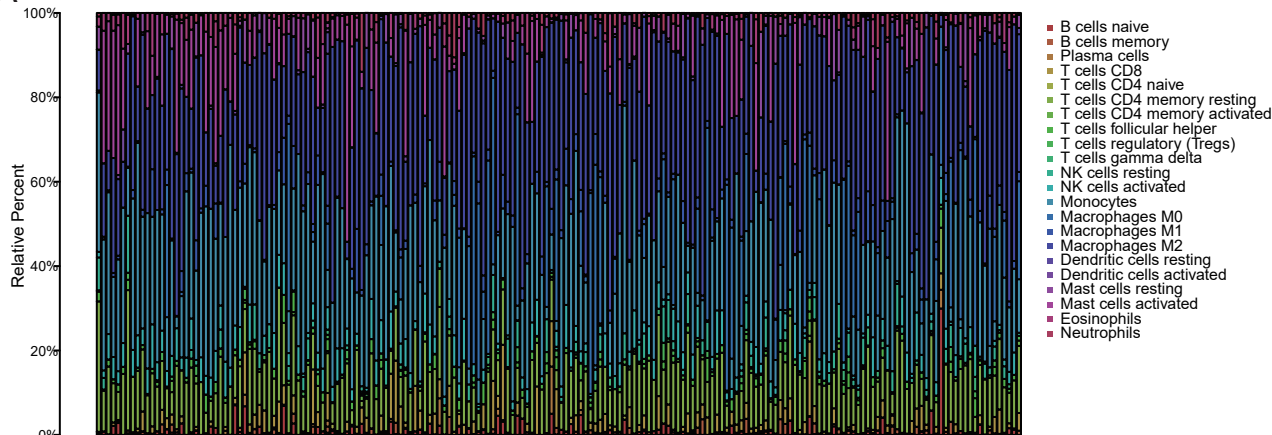

B

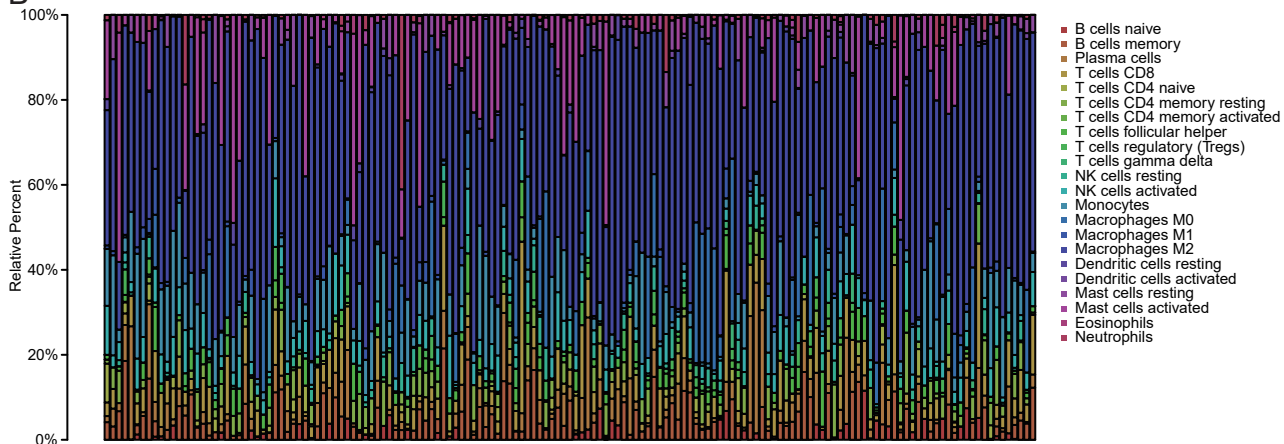

Supplement: Supplementary Materials — Supplementary Figure 1: pan-cancer analysis of HAUS1 based on (A) GTEx and TCGA databases and (B) GEPIA2. Survival curve analysis of HAUS1 expression and (C) DLBC, (D) LIHC, (E) PAAD, and (F) SKCM. Supplementary Figure 2: survival curve analysis of HAUS1 expression and (A) TGCT, (B) THYM. (C) Cox analysis of OS in HAUS1 expression in pan cancer. Supplementary Figure 3: the methylation site of HAUS1 DNA sequence associated with gene expression was revealed using MEXPRESS in (A) GBM and (B) LGG samples. (C) Relationship between HAUS1 expression and four methyltransferases. Red represents DNMT1, blue represents DNMT2, green represents DNMT3a, and purple represents DNMT3b. (D) Correlation between HAUS1 expression and five MMR genes (EPCAM, PMS2, MLH1, MSH2, and MSH6). Supplementary Figure 4: the landscape of 22 types of tumour-infiltrating lymphocytes in glioma samples in (A) TCGA and (B) CGGA datasets. Supplementary Figure 5: predictive powers for glioma infiltration between (A) HAUS1 and common markers, such as (B) PD-L1, (C) CTLA-4, and (D) Siglec15. [file 8027686.f1.zip › 8027686.f1/Supplementary Figure 4 (1).pdf]

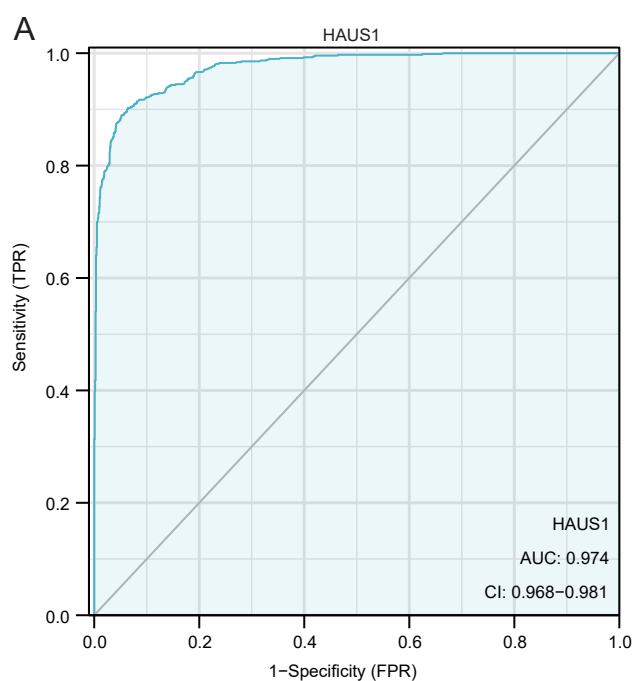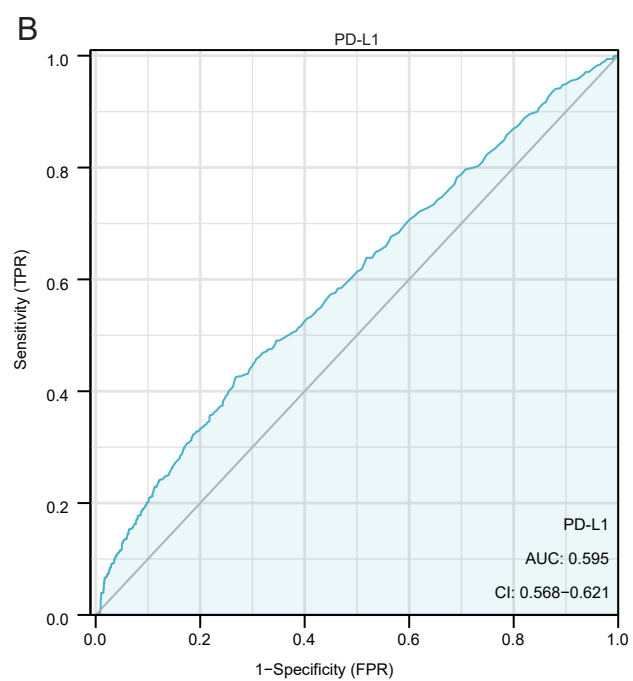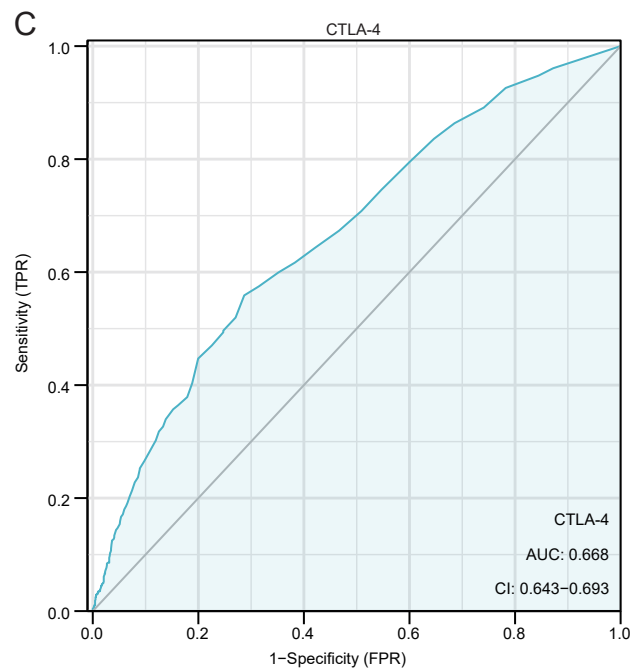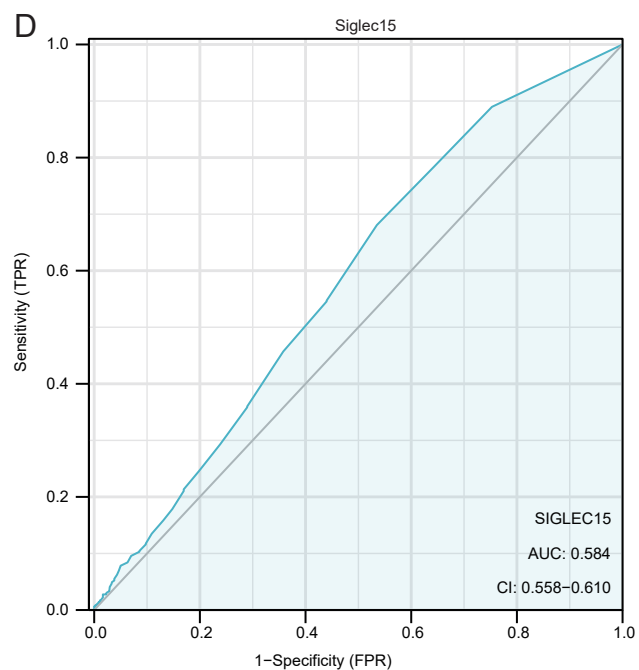

Supplement: Supplementary Materials — Supplementary Figure 1: pan-cancer analysis of HAUS1 based on (A) GTEx and TCGA databases and (B) GEPIA2. Survival curve analysis of HAUS1 expression and (C) DLBC, (D) LIHC, (E) PAAD, and (F) SKCM. Supplementary Figure 2: survival curve analysis of HAUS1 expression and (A) TGCT, (B) THYM. (C) Cox analysis of OS in HAUS1 expression in pan cancer. Supplementary Figure 3: the methylation site of HAUS1 DNA sequence associated with gene expression was revealed using MEXPRESS in (A) GBM and (B) LGG samples. (C) Relationship between HAUS1 expression and four methyltransferases. Red represents DNMT1, blue represents DNMT2, green represents DNMT3a, and purple represents DNMT3b. (D) Correlation between HAUS1 expression and five MMR genes (EPCAM, PMS2, MLH1, MSH2, and MSH6). Supplementary Figure 4: the landscape of 22 types of tumour-infiltrating lymphocytes in glioma samples in (A) TCGA and (B) CGGA datasets. Supplementary Figure 5: predictive powers for glioma infiltration between (A) HAUS1 and common markers, such as (B) PD-L1, (C) CTLA-4, and (D) Siglec15. [file 8027686.f1.zip › 8027686.f1/Supplementary Figure 5 (1).pdf]
