# Supplementary material for: HAUS Augmin-Like Complex Subunit 1 Influences Tumour Microenvironment and Prognostic Outcomes in Glioma
Source: J Oncol. 2022 Jul 12;2022:8027686. doi: 10.1155/2022/8027686 (PMC9296284; doi:10.1155/2022/8027686)
Supplement: Supplementary Materials — Supplementary Figure 1: pan-cancer analysis of HAUS1 based on (A) GTEx and TCGA databases and (B) GEPIA2. Survival curve analysis of HAUS1 expression and (C) DLBC, (D) LIHC, (E) PAAD, and (F) SKCM. Supplementary Figure 2: survival curve analysis of HAUS1 expression and (A) TGCT, (B) THYM. (C) Cox analysis of OS in HAUS1 expression in pan cancer. Supplementary Figure 3: the methylation site of HAUS1 DNA sequence associated with gene expression was revealed using MEXPRESS in (A) GBM and (B) LGG samples. (C) Relationship between HAUS1 expression and four methyltransferases. Red represents DNMT1, blue represents DNMT2, green represents DNMT3a, and purple represents DNMT3b. (D) Correlation between HAUS1 expression and five MMR genes (EPCAM, PMS2, MLH1, MSH2, and MSH6). Supplementary Figure 4: the landscape of 22 types of tumour-infiltrating lymphocytes in glioma samples in (A) TCGA and (B) CGGA datasets. Supplementary Figure 5: predictive powers for glioma infiltration between (A) HAUS1 and common markers, such as (B) PD-L1, (C) CTLA-4, and (D) Siglec15. [file Supplementary Figure 3 (1).pdf › 8027686.f1/Supplementary Figure 3 (1).pdf]

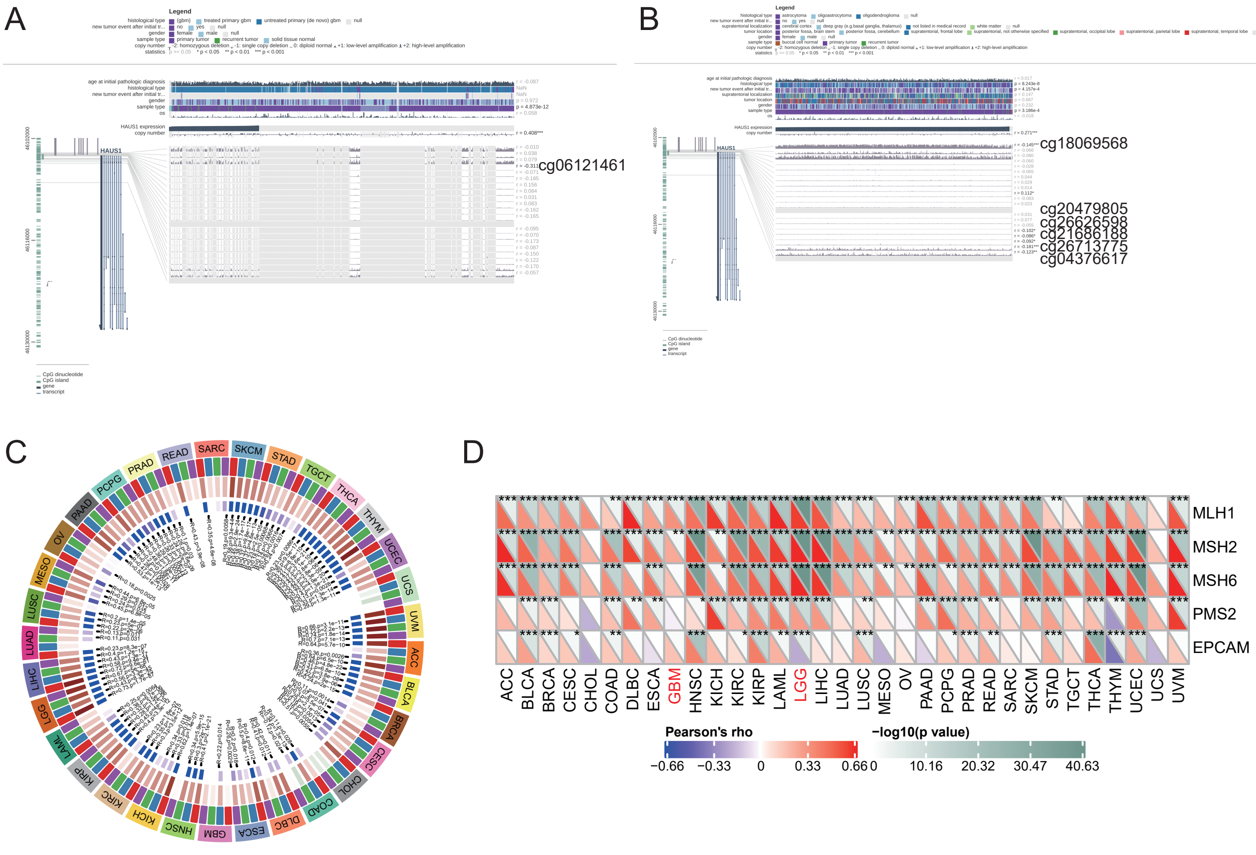

A
B
C
D
Legend
histological type
new tumor event after initial tr...
gender
sample type
copy number
statistics
age at initial pathologic diagnosis
HAUS1 expression
HAUS1
cg06121461
cg18069568
cg20479805
cg26626598
cg21686188
cg26713775
cg04376617
CpG dinucleotide
CpG island
gene
transcript
MLH1
MSH2
MSH6
PMS2
EPCAM
ACC
BLCA
BRCA
CESC
CHOL
COAD
DLBC
ESCA
GBM
HNSC
KICH
KIRC
KIRP
LAML
LGG
LIHC
LUAD
LUSC
MESO
OV
PAAD
PCPG
PRAD
READ
SARC
SKCM
STAD
TGCT
THCA
THYM
UCEC
UCS
UVM
Pearson's rho
−0.66 −0.33 0 0.33 0.66
−log10(p value)
0 10.16 20.32 30.47 40.63
